# Hepatic Wnt1 Inducible Signaling Pathway Protein 1 (WISP-1/CCN4) Associates with Markers of Liver Fibrosis in Severe Obesity

**DOI:** 10.3390/cells10051048

**Published:** 2021-04-29

**Authors:** Olga Pivovarova-Ramich, Jennifer Loske, Silke Hornemann, Mariya Markova, Nicole Seebeck, Anke Rosenthal, Frederick Klauschen, José Pedro Castro, René Buschow, Tilman Grune, Volker Lange, Natalia Rudovich, D. Margriet Ouwens

**Affiliations:** 1 Research Group Molecular Nutritional Medicine, Department of Molecular Toxicology, German Institute of Human Nutrition Potsdam-Rehbruecke, 14558 Nuthetal, Germany; jenniferloske@googlemail.com; 2 Department of Clinical Nutrition, German Institute of Human Nutrition Potsdam-Rehbrücke (DIfE), 14558 Nuthetal, Germany; silkehornemann@o2mail.de (S.H.); maria.markova@yahoo.de (M.M.); seebeck.ni@gmail.com (N.S.); Natalia.rudovich@spitalbuelach.ch (N.R.); 3 Department of Endocrinology, Diabetes and Nutrition, Campus Benjamin Franklin, Charité-Universitätsmedizin Berlin, 12203 Berlin, Germany; 4 German Center for Diabetes Research (DZD), 85764 Munich-Neuherberg, Germany; scientific.director@dife.de (T.G.); Margriet.Ouwens@ddz.de (D.M.O.); 5 Institute of Nutritional Science, University of Potsdam, 14558 Nuthetal, Germany; 6 Clinic for Nutritional Medicine, 10625 Berlin, Germany; Anke@dr-rosenthal.com; 7 Institute of Pathology, Charité-Universitätsmedizin Berlin, Campus Mitte, 10117 Berlin, Germany; frederick.klauschen@charite.de; 8 German Cancer Consortium (DKTK), Partner Site Berlin, German Cancer Research Center (DKFZ), 69120 Heidelberg, Germany; 9 Division of Genetics, Department of Medicine, Brigham and Women’s Hospital and Harvard Medical School, Boston, MA 02115, USA; jose.castro@i3s.up.pt; 10 Aging and Aneuploidy Laboratory, IBMC, Instituto de Biologia Molecular e Celular, Universidade do Porto, 4200-135 Porto, Portugal; 11 i3S, Instituto de Investigação e Inovação em Saúde, Universidade do Porto, 4200-135 Porto, Portugal; 12 Department of Microscopy & Cryo-Electron Microscopy, Max Planck Institute for Molecular Genetics, 14195 Berlin, Germany; buschow@molgen.mpg.de; 13 Department of Molecular Toxicology, German Institute of Human Nutrition Potsdam-Rehbruecke (DIfE), 14558 Nuthetal, Germany; 14 German Center for Cardiovascular Research (DZHK), 13347 Berlin, Germany; 15 Centre for Obesity and Metabolic Surgery, Vivantes Hospital, 13509 Berlin, Germany; Volker.Lange2@helios-gesundheit.de; 16 Helios Klinikum Berlin-Buch, 13125 Berlin, Germany; 17 Division of Endocrinology and Diabetology, Department of Internal Medicine, Spital Bülach, 8180 Bülach, Switzerland; 18 German Diabetes Center, 40225 Duesseldorf, Germany; 19 Department of Endocrinology, Ghent University Hospital, 9000 Ghent, Belgium

**Keywords:** WISP-1/CCN4, CCN proteins, adipokine, obesity, liver, fibrosis, inflammation, hepatic stellate cells

## Abstract

Liver fibrosis is a critical complication of obesity-induced fatty liver disease. Wnt1 inducible signaling pathway protein 1 (WISP1/CCN4), a novel adipokine associated with visceral obesity and insulin resistance, also contributes to lung and kidney fibrosis. The aim of the present study was to investigate the role of CCN4 in liver fibrosis in severe obesity. For this, human liver biopsies were collected from 35 severely obese humans (BMI 42.5 ± 0.7 kg/m^2^, age 46.7 ± 1.8 y, 25.7% males) during bariatric surgery and examined for the expression of CCN4, fibrosis, and inflammation markers. Hepatic stellate LX-2 cells were treated with human recombinant CCN4 alone or in combination with LPS or transforming growth factor beta (TGF-β) and examined for fibrosis and inflammation markers. CCN4 mRNA expression in the liver positively correlated with BMI and expression of fibrosis markers COL1A1, COL3A1, COL6A1, αSMA, TGFB1, extracellular matrix turnover enzymes TIMP1 and MMP9, and the inflammatory marker ITGAX/CD11c. In LX-2 cells, the exposure to recombinant CCN4 caused dose-dependent induction of MMP9 and MCP1. CCN4 potentiated the TGF-β-mediated induction of COL3A1, TIMP1, and MCP1 but showed no interaction with LPS treatment. Our results suggest a potential contribution of CCN4 to the early pathogenesis of obesity-associated liver fibrosis.

## 1. Introduction

Liver fibrosis is a critical complication of non-alcoholic fatty liver disease (NAFLD), which is associated with obesity, metabolic syndrome, and type 2 diabetes. NAFLD is defined by excessive triglyceride accumulation in hepatocytes and represents an increasingly prevalent and common liver disease affecting 20–30% of Western countries [1]. In about 25% of subjects with NAFLD, the disease can progress to non-alcoholic steatohepatitis (NASH) that is histologically characterized by hepatocyte ballooning, apoptosis, and chronic inflammation. Furthermore, patients with NASH may develop hepatic fibrosis, which dramatically increases the risk of irreversible cirrhosis, liver failure, and hepatocellular carcinoma [2]. Thus, hepatic fibrosis is an important component of the progression of NAFLD and NASH to cirrhosis and strongly contributes to the disturbance of liver functions. Besides metabolically-induced fibrosis, other chronic liver diseases caused by viral, inflammatory, or toxic liver injury are also accompanied by liver fibrosis [3].

A key feature of liver fibrosis is an increased remodeling of the extracellular matrix (ECM). The ECM is a complex grid consisting of multiple proteins (collagens, glycoproteins, proteoglycans, elastin, etc.) and essentially contributes to the regulation of liver homeostasis [3]. In liver fibrosis, a high ECM turnover results in increased ECM degradation by matrix metalloproteinases and, simultaneously, in increased accumulation of both new and already existing proteins, which is macroscopically described as fibrosis [3]. Major ECM-producing cells in liver fibrosis are hepatic stellate cells (HSCs) which are activated by a metabolically-induced hepatocellular injury and transformed into highly proliferative myofibroblasts, which express and deposit large quantities of ECM components [3,4].

Multiple pathophysiological pathways contribute in general to metabolically-induced hepatic fibrosis progression and, in particular, to HSC activation [5]. In brief, diet-induced lipid overload of liver cells generates lipotoxicity and glucotoxicity. The resulting endoplasmic reticulum and mitochondrial stress induce the formation of reactive oxygen species and a deregulated unfolded protein response, developing apoptosis and liver injury. This leads to the production of proinflammatory cytokines, chemokines, and damage-associated molecular patterns (DAMPs), which upregulate the activation of Kupffer cells and monocyte-derived macrophages, resulting in chronic inflammation and activation of HSCs into myofibroblasts [5]. Further, high levels of transforming growth factor beta (TGF-β) that occur during chronic liver damage result in activation of HSCs and massive hepatocyte cell death, contributing to the promotion of liver fibrosis [6]. Besides the TGF-β signaling pathway, the WNT pathway also plays an active role in mediating fibrosis in the liver and other organs and tissues and therefore represents a promising target for the treatment of liver fibrosis [7].

WNT-inducible signaling pathway protein-1 (WISP-1, also known as CCN4) is a downstream target of the canonical WNT signaling pathway and belongs to the CCN (CTGF/Cyr61/Nov) family of ECM proteins [8]. Under physiological conditions, CCN4 plays an important role in embryonic development, wound healing, and tissue repair [8]. Aberrant CCN4 expression is associated with various pathologies, including osteoarthritis, fibrosis, and cancer [9,10,11,12]. Particularly, an involvement of CCN4 was demonstrated in mouse lung and kidney fibrosis development [12,13] as well as CCL4-induced liver fibrosis [14], increasing the synthesis of extracellular matrix components (ECM) in fibroblasts [11].

CCN4 is expressed in human liver and adipose tissue [15,16], but other tissues can also possibly contribute to its circulating levels. We recently characterized CCN4 as a novel adipokine associated with visceral obesity and insulin resistance [15,16,17]. Indeed, circulating CCN4 levels are increased in subjects with visceral obesity, positively associated with BMI, and downregulated by weight loss [15,16]. Further, circulating CCN4 levels are higher in patients with gestational diabetes [18] and obese subjects with insulin resistance [19]. Cell experiments showed that CCN4 inhibits insulin action in cultured hepatocytes and muscle cells [16] and stimulates differentiation of human macrophages towards a proinflammatory phenotype [15]. Interestingly, circulating CCN4 levels are associated with visceral adipose tissue fibrosis [19]. However, it is unknown whether CCN4 contributes to the early stages of the development of human liver fibrosis associated with obesity and NAFLD.

To clarify this, we examined the association between CCN4 in circulation and its hepatic expression with fibrosis markers in liver samples from severely obese subjects without advanced liver cirrhosis. To study possible underlying mechanisms, we additionally investigated the effects of CCN4 on a model of HSC. Our results suggest a potential contribution of CCN4 in the pathogenesis of obesity-associated liver fibrosis.

## 2. Materials and Methods

### 2.1. Study Design and Sample Collection

Thirty-five severely obese subjects with an indication for bariatric surgery were recruited for the study in the Berlin-Brandenburg area in Germany from January 2016 to June 2017. The inclusion criteria were (i) a BMI > 40 kg/m^2^ or (ii) a BMI > 35 kg/m^2^ and obesity-related co-morbidities (type 2 diabetes, hypertension, dyslipidemia, obstructive sleep apnea syndrome). Patients suffering from severe infectious diseases, cancer, liver cirrhosis, or alcohol abuse were excluded. Anthropometric measurements were conducted 3–5 days before surgery. Fasting blood sampling was performed on the day of the surgery, while liver biopsies from the left lobe were collected during the bariatric surgery. Body composition was determined by air displacement plethysmography using BOD POD (COSMED, Rome, Italy). Intrahepatic lipid content (IHL) was measured by localized 1H-MRS on a 1.5-T whole-body scanner (MAGNETOM Avanto, Siemens Healthineers, Germany) at the Ernst von Bergmann Hospital in Potsdam. Due to technical restrictions for MR examinations, only a sub-group of 12 subjects could undergo these measurements. Therefore, we additionally assessed triglyceride content in liver samples as described below.

The bariatric surgery took place at the Vivantes Hospital Spandau (Berlin, Germany). The trial was approved by the Ethics Committee of the Charité University Medicine in Berlin (Application No. EA4/006/15), and was conducted in accordance with the Declaration of Helsinki, and registered at www.drks.de accessed on 25 April 2021 (DRKS00009509). All participants provided written informed consent before entering the study.

### 2.2. Blood Sampling and Tissue Collection

On the day of the surgery, blood was sampled, and liver biopsies from the left lobe were collected during the bariatric surgery. A small part of the liver biopsies was stored in 4% formaldehyde for histological analysis, and the rest was flash-frozen in liquid nitrogen and stored at −80 °C until further examinations. Venous blood samples were immediately centrifuged and frozen at −80 °C until analysis.

### 2.3. Histology and Immunohistochemistry

After the liver tissue was immersed in 4% formaldehyde for 24 h at room temperature and embedded in paraffin, it was sectioned in 2 µm histological slices and stained with hematoxylin and eosin (H&E). All of the images were acquired with a BX46 Upright microscope (Olympus, Tokyo, Japan) and were assessed at 20× magnification by an experienced pathologist. Each biopsy was scored on the grade of steatosis, lobular inflammation, hepatocellular ballooning, and fibrosis which were evaluated semi-quantitatively: steatosis (0–3), lobular inflammation (0–2), hepatocellular ballooning (0–2), and fibrosis (0–4) [20]. In particular, the stage of fibrosis was assessed as follows: stage 0: none; stage 1:1a or 1b perisinusoidal zone 3 or 1c portal fibrosis; stage 2: perisinusoidal and periportal fibrosis without bridging; stage 3: bridging fibrosis; and stage 4: cirrhosis [20]. The NAFLD Activity Score (NAS) was determined as the sum of steatosis, hepatocellular ballooning, and lobular inflammation scores, and study subjects were categorized as having no NAFLD (NAS = 0), NAFLD (NAS = 1–2), or NASH (NAS ≥ 3).

To determine hepatic fibrosis, alpha-smooth muscle actin (αSMA, antibody dilution 1:500, Abcam, Berlin, Germany), Sirius red and trichrome stainings were additionally performed by histochemical and immunohistochemical methods, and images were taken with the Axioplan 2 microscope, AxioCam color HRC, and 10× objective Plan-Neofluar (Zeiss, Jena, Germany) (Supplementary Figure S1). Image quantification was performed by the Intellesis Trainable Segmentation Software (ZEN 2.5 System Blue edition) using artificial intelligence. The software was trained manually by outlining the classes of interest as background, stained cytoplasm, nuclei and region of interest (Supplementary Figure S2). The resulting masks were implemented as image analysis templates for the assessment of region parameters in all images (10 images/section). Macrophage infiltration was assessed by CD68 immunohistochemistry (antibody dilution 1:100, Dako/Agilent, Hamburg, Germany), and macrophages were counted in 10 different images per section.

### 2.4. Analytical Procedures

Routine markers were measured in serum using ABX Pentra 400 (HORIBA, Kyoto, Japan). Capillary blood glucose concentrations were measured using a glucose oxidase method on a Dr. Müller Super GL (Dr. Müller Glucose Analyzer, Freital, Germany). WISP-1/CCN4 levels were measured by human direct sandwich WISP-1/CCN4 DuoSet ELISA kit (DY1627; R&D Systems, Germany) in combination with bovine serum albumin (A7030, Sigma, Munich, Germany) or human serum albumin (A1887, Sigma) and performed on 96-well high-binding assay plates (82.1581, Sarstedt, Nümbrecht, Germany) as described in [17]. Commercially available ELISA kits were used for the measurements of serum/plasma insulin (Insulin ELISA, Mercodia AB, Uppsala, Sweden) and TIMP1 (all from R&D Systems, Minneapolis, MN, USA), and the U-Plex assay was used to measure IL6, tumor necrosis factor alpha (TNFα), and MCP1 (MSD, Rockville, MD, USA).

Analysis of hepatic triglyceride content was performed according to the Triglyceride Determination Kit (Sigma Aldrich Chemie, Steinheim, Germany), and the absorbance changes were detected at 540 nm by spectrophotometry.

### 2.5. Cell Culture

The immortalized human hepatic cell-line LX-2 (Merck, Cat. # SCC064) was cultured in a DMEM High Glucose-Medium (Gibco/Invitrogen, Karlsruhe, Germany), supplemented with 2% fetal calf serum and 1% antibiotic-antimycotic (both from Sigma Aldrich, Munich, Germany). For the experiment, cells were seeded at a density of 300,000 cells/well in 6 well plates (TPP) and treated with human recombinant WISP-1 (10, 100, 500 ng/mL; PeproTech, Hamburg, Germany) alone or in combination with 1 ng/mL lipopolysaccharides (LPS) (Sigma Aldrich, Munich, Germany) or 1 ng/mL TGF-β (PeproTech) for 24 h. Cell culture supernatants were analyzed using the Bio-Rad 200 System (Bio-Rad, Feldkirchen, Germany) and ProcartaPlex multiplex immunoassay (Invitrogen/Affymetrix, Darmstadt, Germany).

### 2.6. Gene Expression Analyses

Total RNA from liver and cell samples was purified using the RNeasy Mini Kit (both from Qiagen, Hilden, Germany) or the NucleoSpin^®^ RNA II kit (MACHEREY-NAGEL, Düren, Germany), respectively. RNA concentration was measured using an ND-1000 spectrophotometer (Nanodrop, PeqLab, Erlangen, Germany). Single-stranded cDNA was synthesized with a High-Capacity cDNA Reverse Transcription kit (Thermo Fisher Scientific, Darmstadt, Germany). Quantitative real-time PCR (qPCR) was performed by the ViiA 7 sequence detection system using Power SYBR Green PCR Master Mix (Applied Biosystems, Forster City, CA, USA) and specific primers as described in [21]. Gene expression was assessed by the standard curve method and normalized to the reference gene beta-glucuronidase (GUSB). Primer sequences are shown in Supplementary Table S1.

### 2.7. Analysis of WISP1 Expression in Human Tissue Panel

We used the publicly available data GTEx v7 from dbGap (https://www.gtexportal.org accessed on 25 April 2021) and analyzed the mRNA expression of WISP1 in 48 human tissues, both in males and females as described in [22]. The data used for the analyses described in this manuscript were obtained from dbGaP accession number phs000424.v8.p2 on the 28 January 2019.

### 2.8. Statistical Analyses

Statistical analyses were performed with SPSS v.20 (SPSS, Chicago, IL, USA) as described in [21]. All data are reported as mean ± standard error of the mean (SEM). Data distribution was determined by the Shapiro–Wilk test. Variables showing a skewed distribution were log-transformed prior to analysis. A paired or two-sample *t*-test and Mann-Whitney *U* test were used to compare the two groups, while an ANOVA with subsequent post hoc analysis was applied to compare more than two groups. A correlation analysis was performed through the Pearson’s coefficient or the Spearman’s rank correlation coefficient depending on data distribution. The index of whole-body insulin resistance (HOMA-IR) was calculated as: fasting insulin (µU/mL) × fasting glucose in (mM)/22.5. Statistical significance was defined as *p* < 0.05.

## 3. Results

### 3.1. Circulating CCN4 and Anthropometric and Biochemical Parameters

The analysis of CCN4 in 48 human tissues revealed its mRNA expression in most investigated organs, including lung, kidney, heart, adipose tissue, and liver, and showed no difference between male and female subjects (Supplementary Figure S3). Because CCN4 can be secreted in the circulation and was recently described to play a role in the pathogenesis of obesity [15], we further investigated the association of its circulating levels with anthropometric and biochemical parameters as well as markers of hepatic function.

For this, we analyzed blood samples from 35 severely obese subjects without advanced liver cirrhosis (9 men and 26 women with a BMI of 42.5 kg/m^2^, and a fat mass of 53.3% of their body weight) (Table 1). Three subjects had type 1 diabetes, and eight subjects had type 2 diabetes. Histological analysis of liver biopsies showed that 26 subjects had liver steatosis, 10 subjects had ballooning, 28 subjects displayed lobular inflammation, and 24 subjects showed hepatic fibrosis. According to the NAS scoring system [20], 11 subjects had non-alcoholic steatohepatitis (NASH). Within the NASH group, all participants had steatosis: ten subjects had lobular inflammation, eight subjects showed ballooning, and nine subjects showed fibrosis. The mean circulating CCN4 level was 76.7 ± 15.0 pg/mL. The blood sample of one subject was missing, and in 14 subjects (8 without NASH and 6 with NASH), circulating CCN4 levels were below the limit of detection. There were no differences in circulating CCN4 levels between subjects with (88.9 ± 20.1 pg/mL; *n* = 15) and without (73.8 ± 19.1 pg/mL; *n* = 5) NASH and between subjects with different fibrosis scores and NAS scores assessed histologically (Supplementary Figure S4). A correlation analysis revealed a positive association between circulating CCN4 levels and fasting glucose (*r* = 0.480. *p* = 0.032) but not BMI, fasting insulin, HOMA-IR, liver enzymes (ALT, AST, GGT), NAFLD liver fat score [23], triglyceride levels assessed in liver samples, and serum cytokines (IL6, TNF alpha, MCP1).

### 3.2. Association of Hepatic CCN4 Expression with Fibrosis Markers

**Figure 1:**
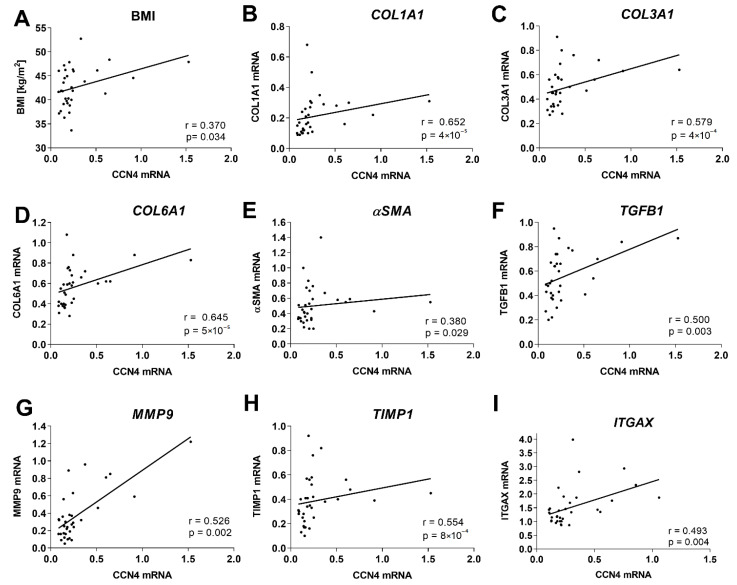
Association of CCN4 with fibrosis markers in human liver. Correlations of CCN4 mRNA expression with (**A**) BMI, (**B**) COL1A1, (**C**) COL3A1, (**D**) COL6A1, (**E**) αSMA, (**F**) TGFB1, (**G**) MMP9, (**H**) TIMP1 and (**I**) ITGAX (*n* = 35).

To investigate the association between CCN4 and fibrosis markers in the liver, mRNA expression was determined using qPCR in a total of 33 samples. All samples showed histological hepatic fibrosis scores between 0 and 2, i.e., no to moderate liver fibrosis (Supplementary Figure S1). Hepatic CCN4 expression positively correlated with BMI (*r* = 0.370, *p* = 0.034) (Figure 1A) but showed no relation to the presence of NASH, NAS score, or hepatic triglyceride content (Supplementary Figure S5). We found a positive correlation between CCN4 expression and mRNA expression of fibrosis markers—three collagen genes COL1A1 (*r* = 0.652; *p* < 0.001), COL3A1 (*r* = 0.579; *p* < 0.001), COL6A1 (*r* = 0.645; *p* < 0.001), αSMA, (*r* = 0.380; *p* = 0.029), as well as TGFB1 (*r* = 0.500, *p* = 0.003), a key regulator of tissue fibrosis (Figure 1B–F), but not with αSMA, Sirius red and trichrome stainings quantified by an automated histological image analysis. In addition, TIMP1 (*r* = 0.554; *p* < 0.001) and MMP9 (*r* = 0.526; *p* < 0.001), two important enzymes in the regulation of ECM turnover, were positively associated with hepatic mRNA expression of CCN4 (Figure 1G–H) and other fibrosis markers (Supplementary Table S2). CCN4 mRNA levels also showed a tendency to associate with the macrophage marker ITGAX (CD11c) (*r* = 0.304, *p* = 0.091) (Figure 1I), but not with other hepatic cytokine markers (IL6, TNFα, MCP1, IL1B, IL10) or with macrophage numbers in liver sections.

We also observed no correlation between hepatic CCN4 expression and key determinants of ER-stress (BiP, XBP1s, XBP1, DDIT3), fatty acid β-oxidation (MCAD, ACOX1, ACOX2, CPT1A, PPARA), lipid storage (PPARG, SCD1), and *de novo* lipogenesis (ChREBP, FASN, ACC1, ACC2, AMPK) except for SREBP1c (*r* = 0.461, *p* = 0.007).

### 3.3. Effects of CCN4 Treatment in Human Hepatic Stellate Cells

**Figure 2:**
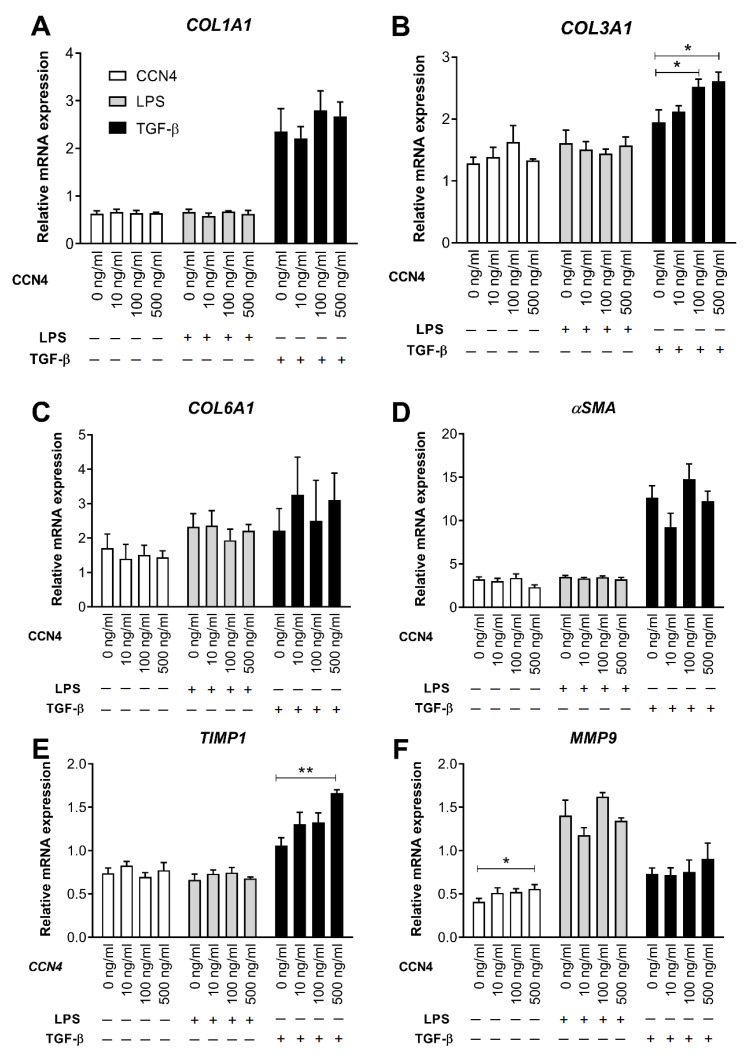
CCN4 effects on fibrosis marker expression in hepatic stellate LX-2 cells. LX-2 cells were stimulated for 24 h with human recombinant CCN4 (0, 10, 100 or 500 ng/mL; in white) alone or in combination with 1 ng/mL LPS (grey bars) or 1 ng/mL TGF-β (black bars), and the mRNA expression of *COL1A1* (**A**), *COL3A1* (**B**), *COL6A1* (**C**), *αSMA* (**D**), *TIMP1* (**E**), and *MMP9* (**F**) was measured by qPCR (*n* = 4). Data are shown as mean ± SEM. * *p* < 0.05, ** *p* < 0.01.

**Figure 3:**
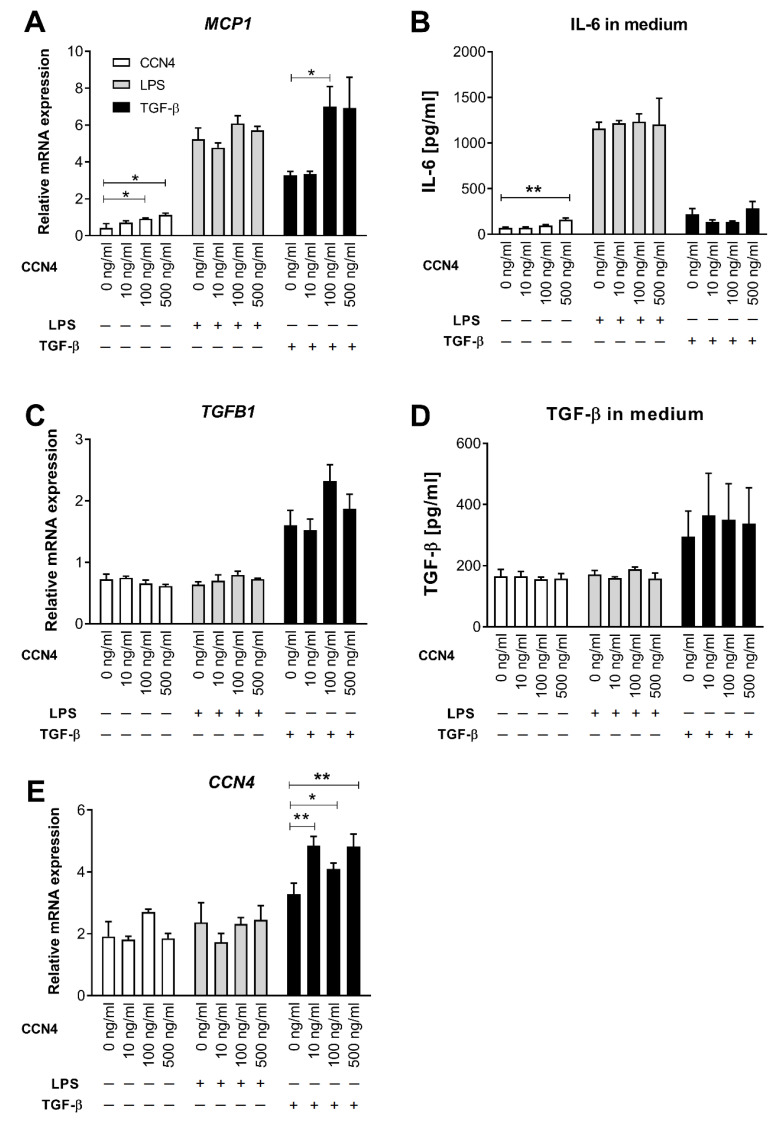
CCN4 effects on inflammatory markers in hepatic stellate LX-2 cells. LX-2 cells were stimulated for 24 h with human recombinant CCN4 (0, 10, 100 or 500 ng/mL; in white) alone or in combination with 1 ng/mL LPS (grey bars) or 1 ng/mL TGF-β (black bars) (*n* = 4). (**A**,**C**,**E**) Gene expression was measured by qPCR. (**B**,**D**) Cytokine secretion was determined by multiplex assay. Data are shown as mean ± SEM. * *p* < 0.05, ** *p* < 0.01.

HSCs play a key role in the initiation and progression of liver fibrosis by secreting fibrogenic factors that encourage hepatic fibrocytes, fibroblasts, and bone marrow-derived myofibroblasts to produce collagen [24]. To investigate whether HSC activation represents a link between CCN4 and liver fibrosis, we investigated the effects of CCN4 on LX-2 cells, a well-established model for the study of HSC [25]. For this, LX-2 cells were treated with human recombinant CCN4 (10, 100, 500 ng/mL) alone or in combination with 1 ng/mL LPS or 1 ng/mL TGF-β for 24h. TGF-β treatment strongly increased the mRNA expression of fibrosis markers COL1A1, COL3A1, αSMA, and TIMP1 (Figure 2), whereas LPS upregulated MMP9 and cytokines MCP1, IL6 (Figure 3A,B), and IL1β (data not shown). MMP9, MCP1 expression, and IL-6 secretion were upregulated after CCN4 stimulation alone. CCN4 did not show an additive effect on LPS stimulation, but an increase in COL3A1, TIMP1, and MCP1 mRNA in combination with TGF-β was observed (Figure 2A,E and Figure 3A). Notably, CCN4 treatment affected neither TGFB1 mRNA nor secretion levels (Figure 3C,D). On the contrary, all tested TGF-β concentrations increased CCN4 expression in LX-2 cells, and CCN4 upregulated its own expression in combination with TGF-β (Figure 3E). Thus, our findings on stellate cells highlighted a role for CCN4 in the induction of liver fibrosis.

## 4. Discussion

Our study provided the first piece of evidence that the novel adipokine WISP1/CCN4, which is increased in visceral obesity, might contribute to the early development of obesity-associated fibrosis even before marked cirrhotic changes occur. We showed for the first time that, in severely obese subjects without advanced liver cirrhosis, hepatic CCN4 expression is associated with BMI, expression of fibrosis markers, and ECM turnover enzymes in liver samples. In contrast, circulating CCN4 was not associated with liver fibrosis. Further, in the HSC model, exposure to CCN4 caused a dose-dependent induction of fibrosis markers and proinflammatory cytokines alone or in combination with TGF-β treatment. These findings extend our previous studies showing the functions of CCN4 in insulin resistance and inflammation [15,16,17,26].

We and others detected CCN4 expression in both human and mouse liver and adipose tissue [15,16]. GTEx data, as well as our findings, showed that CCN4 levels are more abundant in visceral adipose tissue (VAT) than in subcutaneous adipose tissue (SAT) and the liver [16]. Furthermore, feeding mice a high-fat diet increases CCN4 mRNA levels in both the liver and adipose tissue [15]. Finally, circulating CCN4 levels and CCN4 expression in VAT are higher in subjects with visceral obesity compared to non-obese subjects [16].

CCN4 can be secreted into the circulation from adipocytes but is not produced in monocytes and macrophages [15]. We, therefore, postulate that VAT is the main source of circulating CCN4, at least in visceral obesity, although the liver and other organs could also contribute to the circulating CCN4 levels. Circulating CCN4 might affect the metabolic regulation in other organs, including the liver. In vitro experiments showed that CCN4 could inhibit insulin action in hepatocytes and muscle cells by affecting Akt/FOXO signaling [16] and suggested that CCN4 might induce insulin resistance in the liver and muscle. CCN4 can also activate the TLR4/JNK signaling pathway [27], linking CCN4-induced insulin resistance with inflammation. In human macrophage cultures, CCN4 treatment induced the expression and secretion of proinflammatory cytokines IL-6, TNFα, and IL-1β and shifted cell differentiation towards the proinflammatory M1 type [15], acting via the CD14–TLR4 signaling [28]. In our previous work, *CCN4* mRNA expression in VAT showed a positive correlation with *CCL2* expression in a cohort of normal-weight and obese subjects [16]. In a cohort of subjects with type 2 diabetes, the circulating CCN4 levels correlated positively with fat mass, serum leptin, resistin, and visfatin levels [26], suggesting that CCN4 may contribute to the metabolically-induced tissue inflammation.

Unexpectedly, in the present study, circulating CCN4 showed no association with markers of liver function such as liver enzymes, SAF score, and fibrosis score and was not different in subjects with or without NASH. As mentioned above, other organs besides the liver and adipose tissue can also contribute to the circulating levels of CCN4 and could explain the absence of correlation between circulating CCN4 and local fibrosis.

Nevertheless, in the liver, CCN4 mRNA expression was associated with BMI and expression of fibrosis markers COL1A1, COL3A1, COL6A1, αSMA, TGFB1, ECM degradation enzyme MMP9, the inhibitor of matrix metalloproteinases TIMP1, and inflammatory marker ITGAX/CD11c. We, therefore, hypothesize that, in the liver of severely obese subjects, CCN4 secreted into the ECM acts locally in an autocrine or paracrine fashion resulting in the upregulated fibrosis and inflammation. However, we cannot exclude that circulating CCN4 also contributes to these processes. Other research groups showed that CCN4 mediates pulmonary fibrosis in mice and is upregulated in humans with idiopathic pulmonary fibrosis [13]. CCN4 was also shown to be associated with renal fibrosis in a TGF-β-induced tubular epithelial cell model, a mouse model of obstructive nephropathy, and in subjects with chronic kidney disease [12]. Several studies showed that the other CCN family member, connective tissue growth factor (CTGF/CCN2), facilitates liver fibrosis in humans and animal models [29,30], whereas CYR61/CCN1, in contrast, promotes regression of liver fibrosis through induction of cellular senescence in hepatic myofibroblasts [31].

Interestingly, hepatic CCN4 expression was not associated with expression levels of the key genes of the lipid metabolism except for SREBP1c. This suggests that, in the liver, CCN4 is not markedly involved in the regulation of lipid metabolism and lipid accumulation.

Our data also demonstrated an important role of HSC in the CCN4-mediated regulation of liver fibrosis. We showed in LX-2 cells, a well-established model for the study of HSC [25], that exposure to CCN4 caused induction of fibrosis markers and proinflammatory cytokines alone or in combination with TGF-β treatment. Similar results were demonstrated in a previously published study [32]. Chronic liver injury induced the increased production of TGF-β by a number of non-parenchymal liver cells, including HSCs and immune cells, resulting in the activation of HSCs and excessive ECM protein production accompanied by hepatocyte cell death [6]. Likewise, TGF-β can promote its own expression, which was also observed in our experiments (Figure 3C). Furthermore, TGF-β showed a tendency to increase CCN4 expression (*p* = 0.06). This finding is in agreement with data on CCN2, which drives collagen production in HSC downstream of TGF-β [30]. Finally, CCN4 upregulated its own expression in HSC in combination with TGF-β (Figure 3E), which might accelerate the progression of liver fibrosis via a vicious circle. In general, the interaction between TGF-β and WNT signaling pathways (which is an upstream pathway of CCN4) is complex; it can take place on extracellular, cytoplasmic, and nuclear levels and might depend on the specific tissue and on the pathophysiological situation [33].

Interestingly, CCN4 upregulated the expression of both the fibrosis marker COL3A1 and the ECM turnover enzyme TIMP1 in TGF-β—treated LX-2 culture and also increased the mRNA levels of another ECM turnover enzyme, MMP9. A similar effect of recombinant CCN4 treatment on TIMP1 expression was found on human lung fibroblasts [13]. Moreover, TIMP1 and MMP9 expression positively correlated with hepatic mRNA levels of CCN4 and other fibrosis markers in our association analysis. Thus, our data suggest that CCN4 might stimulate both excessive hepatic production of ECM proteins, such as collagens and αSMA and increased expression of ECM turnover enzymes, which are both increased in the pathogenesis of liver fibrosis [3]. MMPs and TIMPs play a pivotal role in matrix remodeling during hepatic injury and repair [34]. Therefore, TIMP1 increase upon CCN4 treatment could be a protective reaction in response to fibrosis progression.

**Figure 4:**
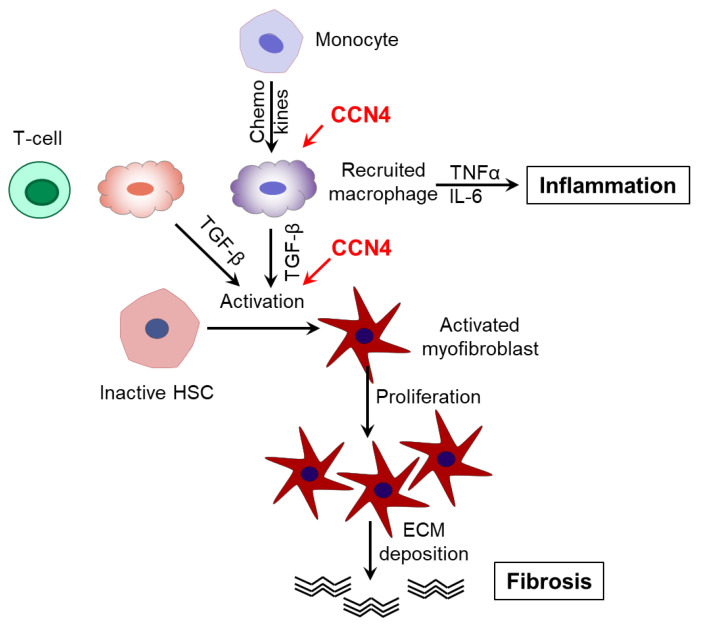
A hypothesized role of CCN4 in the pathogenesis of liver fibrosis. Both immune cells and so-called hepatic stellate cells play a key role in the pathogenesis of liver fibrosis. Upon metabolic disturbances, infiltrated T-cells, macrophages, and other immune cell types, as well as the liver-resident macrophages, induce the activation of HSC, their proliferation, and ECM deposition. CCN4 might accelerate both HSC activation and liver inflammation contributing in this way to the aggravation of liver fibrosis.

Besides the possible direct impact of CCN4 on HCS activation, the next possible mechanism of liver fibrosis might be that CCN4 accelerates liver inflammation and the corresponding production of proinflammatory cytokines and TGF-β by infiltrating macrophages and Kupffer cells which in turn induces HSC activation [35]. In agreement with this, we found a tendency towards a positive association of hepatic CCN4 with inflammatory marker ITGAX and a CCN4-induced upregulation of MCP1 and IL6 in LX2 cells. *MCP1* expression was additionally induced in TGF-β—treated cultures suggesting, again, the tight interaction between TGF-β and WNT/CCN4 signaling pathways. Based on our findings, we suggest that CCN4 might accelerate both HSC activation and liver inflammation contributing, in this way, to the aggravation of liver fibrosis (Figure 4). However, the exact molecular mechanism of the observed CCN4 effects in liver fibrosis still needs to be elucidated. In particular, in vivo experiments using an animal model of obesity-associated fibrosis in both CCN4-deficient and wild-type mice would shed light on underlying mechanisms.

Several limitations of our study have to be mentioned. Firstly, the subject number in our study was relatively small and might not have enough power to detect some associations. For the same reason, and because in some subjects, the circulating WISP1 levels were under the detection limit, we were not able to perform data analysis in subgroups of subjects with and without NASH or diabetes separately. Nevertheless, in the whole cohort, we were able to find a range of significant associations of CCN4 with fibrosis markers and with the BMI suggesting the CCN4 role in liver fibrosis. Secondly, we could only assess the mRNA expression of WISP1 and other markers because no tissue lysates were available for the protein assessment. The quantification of CCN4 and fibrosis markers at the protein level, i.e., by Western blotting, might provide more information about their relationships. Although we provided the fibrosis marker assessment by the automated histological image analysis, we did not find the correlation between CCN4 expression and fibrosis. This might be explained by insufficient sensitivity of the quantification method used or, again, by the small subject number. Thirdly, the study provided only cross-sectional observations giving no possibility to follow up on the CCN4 effects during the progression of obesity or weight loss. However, the collection of repeated human liver samples is extremely difficult, and the investigation of this question looks more realistic in animal studies.

Taken together, our results suggest a contribution of CCN4 to the pathogenesis of obesity-associated liver fibrosis. However, the molecular mechanisms of the relationship between CCN4 and liver fibrosis have to be evaluated in future studies.

## Figures and Tables

**Table 1:** Baseline Anthropometric and Clinical Parameters of Study Participants.

Parameter	Values
*n*	35
Gender (*n*, men/women)	9/26
Age (years)	46.7 ± 1.8
Weight (kg)	124.7 ± 3.9
BMI (kg/m^2^)	42.5 ± 0.7
Fat mass (%)	53.3 ± 1.6
IHL (%) ^a^	13.2 ± 2.4
AST (U/L)	25.5 ± 2.4
ALT (U/L)	31.9 ± 2.2
GGT (U/L)	33.7 ± 5.3
Creatinine (μmol/L)	74.5 ± 4.2
Urea (mmol/L)	5.4 ± 0.9
Uric acid (μmol/L)	315.0 ± 15.5
Cholesterol (mmol/L)	4.15 ± 0.18
HDL-c (mmol/L)	0.96 ± 0.03
LDL-c (mmol/L)	1.90 ± 0.16
Triglyceride (mmol/L)	3.01 ± 0.23
NEFA (mmol/L)	1.85 ± 0.56
Fasting glucose (mmol/L)	7.24 ± 0.43
Fasting insulin (mU/L)	15.3 ± 1.5
HOMA-IR	4.94 ± 0.58
HbA_1c_ (%)	5.9 ± 0.2
Diabetes type 1 (*n*)	3
Diabetes type 2 (*n*)	8
Hepatic steatosis (*n*)	26
Ballooning (*n*)	10
Lobular inflammation (*n*)	28
Hepatic fibrosis (*n*)	24
NASH (*n*)	11

Values are presented as means ± SEM. ^a^—IHL data available for 12 subjects.

## Data Availability

The data presented in this study are available from the corresponding author upon reasonable request.

## Supplementary Materials

The following are available online at https://www.mdpi.com/article/10.3390/cells10051048/s1, Figure S1: Representative histological and immunohistochemical liver images, Figure S2: CCN4 expression in 48 human tissues of male and female subjects, Figure S3: Circulating CCN4 levels in subjects with different liver scores, Figure S4: Hepatic CCN4 mRNA expression levels in subjects with different liver scores, Figure S5: Circulating CCN 4 levels in subjects with different liver steatosis scores Circulating, Table S1: Primers used for real-time PCR, Table S2: Correlation analysis of hepatic fibrosis marker and ECM turnover enzymes.
