# Supplementary material for: Hepatic Wnt1 Inducible Signaling Pathway Protein 1 (WISP-1/CCN4) Associates with Markers of Liver Fibrosis in Severe Obesity
Source: Cells. 2021 Apr 29;10(5):1048. doi: 10.3390/cells10051048 (PMC8146455; doi:10.3390/cells10051048)
Supplement: Supplementary file 1 [file cells-10-01048-s001.zip › Supplement/FigureS1_histo_liver_new.pdf]

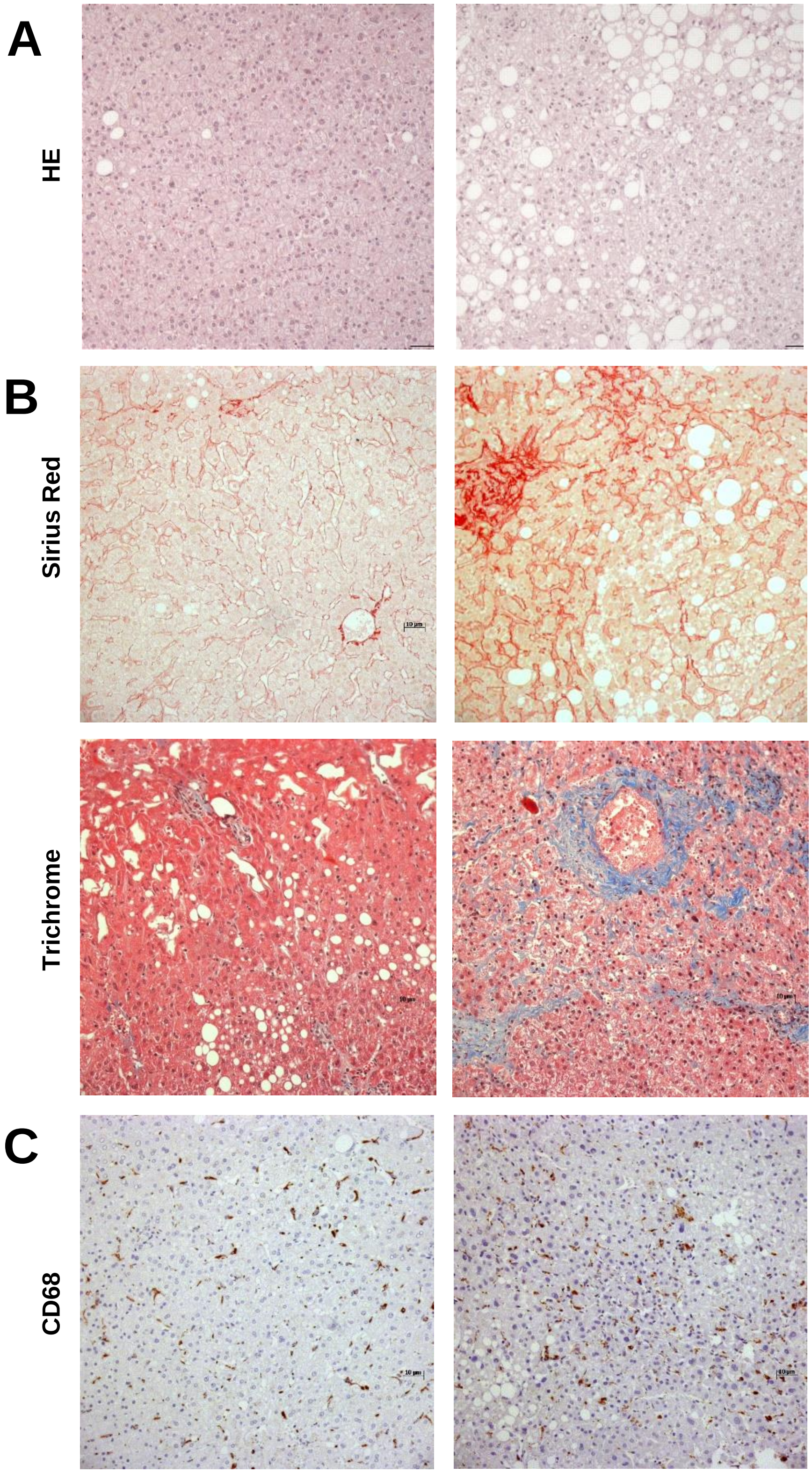


**Supplementary Figure 1: Representative histological and immunohistochemical liver images.** Liver sections were stained with **(A)** hematoxyline-eosine (HE, *left* - healthy liver, *right* – steatosis (score 2)); **(B)** Sirius Red and trichrome used for detection of tissue fibrosis (*left* – no fibrosis (score 0), *right* – moderate fibrosis (score 2)), and **(C)** CD68 antibody used for detection of tissue macrophages (*left* – no inflammation (score 0), *right* – moderate inflammation (score 1)). Images were taken with Axioplan 2 microscope, AxioCam color HRC, 10x objective Plan Neofluar (Zeiss).
