# Supplementary material for: Hepatic Wnt1 Inducible Signaling Pathway Protein 1 (WISP-1/CCN4) Associates with Markers of Liver Fibrosis in Severe Obesity
Source: Cells. 2021 Apr 29;10(5):1048. doi: 10.3390/cells10051048 (PMC8146455; doi:10.3390/cells10051048)
Supplement: Supplementary file 1 [file cells-10-01048-s001.zip › Supplement/FigureS2_histo_liver_Intellesis_R1.pdf]

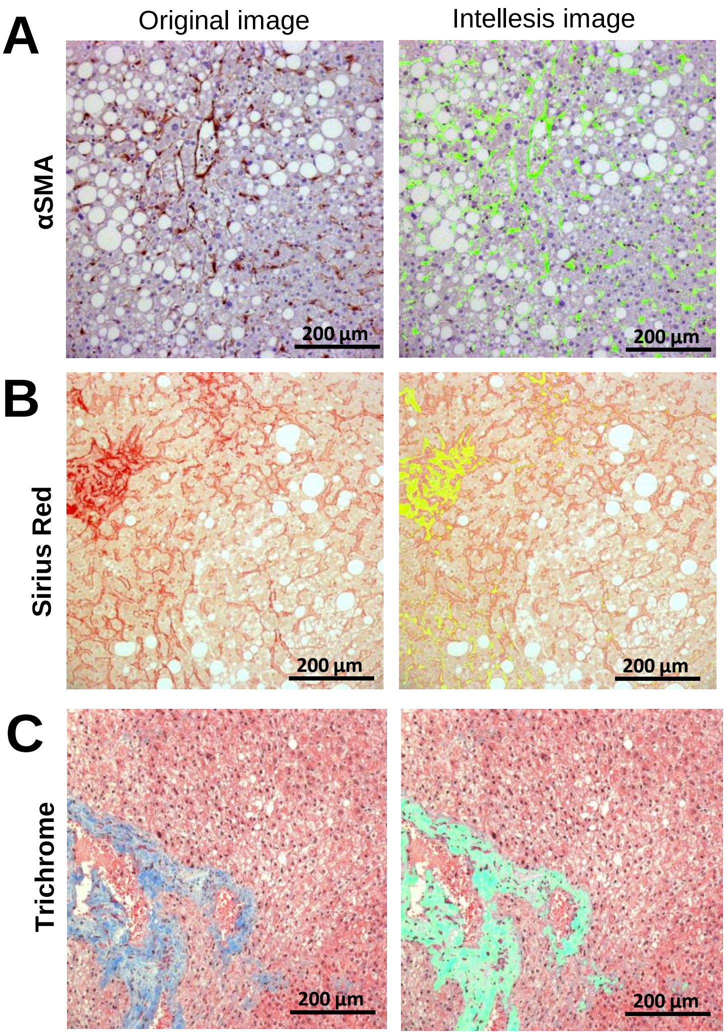

Supplementary Figure 2. Quantification of histological and immunohistochemical images by an automated histological image analysis.

Representative images of liver sections stained with (A) alpha smooth muscle actin (αSMA); (B) Sirius Red, and (C) trichrome (*left* – original image, *right* – images with a mask after applying the Intellesis Software). Images were taken with Axioplan 2 microscope, AxioCam color HRC, 10x objective Plan Neofluar (Zeiss).
