# Supplementary material for: Hepatic Wnt1 Inducible Signaling Pathway Protein 1 (WISP-1/CCN4) Associates with Markers of Liver Fibrosis in Severe Obesity
Source: Cells. 2021 Apr 29;10(5):1048. doi: 10.3390/cells10051048 (PMC8146455; doi:10.3390/cells10051048)
Supplement: Supplementary file 1 [file cells-10-01048-s001.zip › Supplement/FigureS3_CCN4_expr_diff_tissues.pdf]

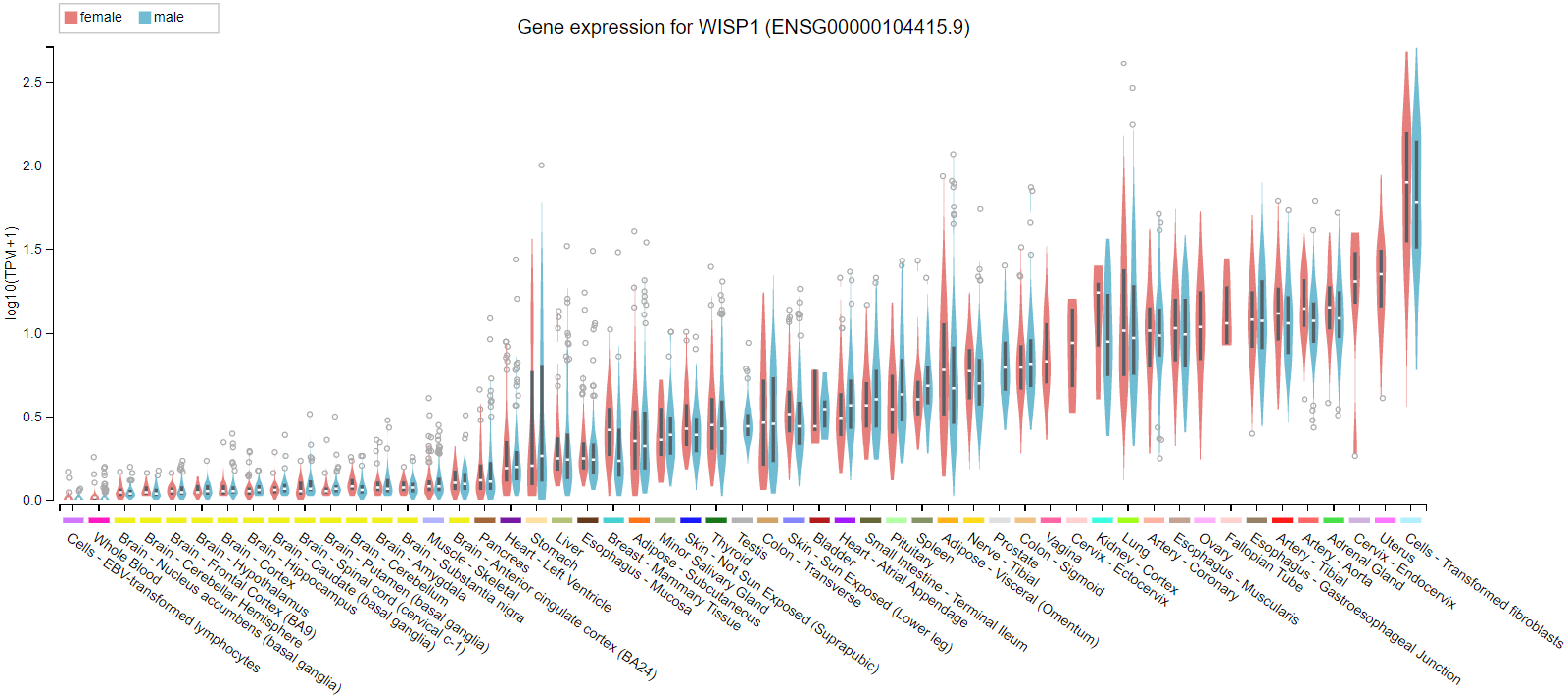

Supplementary Figure 3: CCN4 expression in 48 human tissues of male and female subjects.
Gene expression data was obtained from GTEx data set (https://www.gtexportal.org).
