# Supplementary material for: Hepatic Wnt1 Inducible Signaling Pathway Protein 1 (WISP-1/CCN4) Associates with Markers of Liver Fibrosis in Severe Obesity
Source: Cells. 2021 Apr 29;10(5):1048. doi: 10.3390/cells10051048 (PMC8146455; doi:10.3390/cells10051048)
Supplement: Supplementary file 1 [file cells-10-01048-s001.zip › Supplement/FigureS5_mRNA_CCN4_NAFLD_scores_R1.pdf]

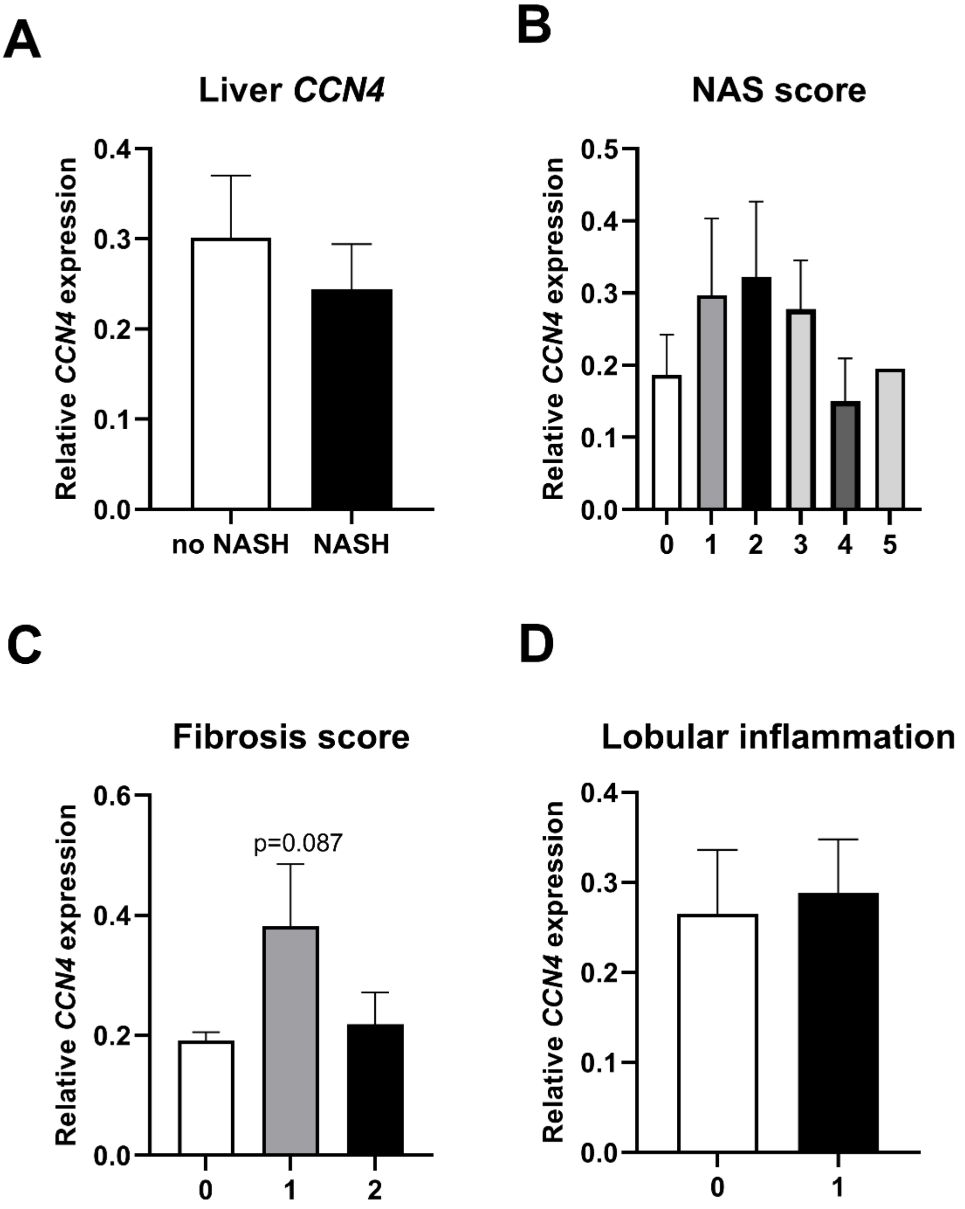

Supplementary Figure 5: Hepatic CCN4 mRNA expression levels in subjects with different liver steatosis scores. Hepatic CCN4 mRNA levels in subjects **(A)** with and without NASH; **(B)** with different NAS score; **(C)** with and without hepatic fibrosis; **(D)** with and without lobular inflammation. Data are shown as mean ± SEM.
