# Supplementary material for: Hepatic Wnt1 Inducible Signaling Pathway Protein 1 (WISP-1/CCN4) Associates with Markers of Liver Fibrosis in Severe Obesity
Source: Cells. 2021 Apr 29;10(5):1048. doi: 10.3390/cells10051048 (PMC8146455; doi:10.3390/cells10051048)
Supplement: Supplementary file 1 [file cells-10-01048-s001.zip › Supplement/Table_S1_primer.pdf]

Supplementary Table 1: Primers used for real-time PCR.

Gene name	Gene symbol	Primer sequence	
		forward	reverse
**Inflammatory genes**			
Interleukin 10	IL10	ACGGCGCTGTCATCGATT	GGCATTCTTCACCTGCTCCA
Interleukin 6	IL6	AGCCCTGAGAAAGGAGACATGTA	TCTGCCAGTGCCTCTTTGCT
Monocyte chemoattractant protein 1	MCP1 (CCL2)	CATAGCAGCCACCTTCATTCC	TCTGCAGTGAGATCTTCCTATTGG
Tumour necrosis factor alpha	TNFα	GGACCTCTCTCTAATCAGCCCTC	TCGAGAAGATGATCTGACTGCC
Interleukin 1 beta	IL1B	GCAATGAGGATGACTTGTTCTTTG	CAGAGGTCCAGGTCCTGGAA
Integrin subunit alpha x	ITGAX (CD11c)	TTTCGAGGAATTCAGGCGC	TGAGGCATGGAACAATCGGT
**Fibrosis genes**			
Alpha smooth muscle actin	αSMA (ACTA2)	TCAATGTCCCAGCCATGTAT	CAGCACGATGCCAGTTGT
Collagen1 alpha 1	COL1A1	ACGAAGACATCCCACCAATC	GGGCAGTTCTTGGTCTCGT
Collagen3 alpha 1	COL3A1	GCCAAATATGTGTCTGTGACTCA	GGGCGAGTAGGAGCAGTTG
Collagen6 alpha 1	COL6A1	ACGAGCTGGTCAAGTTCGAG	AGGCTCTTGATGGCTTCCTT
Transforming growth factor beta 1	TGF-β1	AATTGAGGGCTTTCGCCTTAG	CCGGTAGTGAACCCGTTGAT
Matrix metallopeptidase 9	MMP9	GATGCGTGGAGAGTCGAAAT	CTATCCAGCTCACCGGTCTC
Tissue inhibitor of metalloproteinase 1	TIMP1	TCTCATTGCTGGAAAACTGC	AAACAGGGAAACACTGTGCAT
WNT1-inducible-signaling pathway protein 1	WISP-1	GGCCAGGCTCTTCCTTGAAT	TAGAGGAGTGTTCCAGGGCA
**Housekeeping genes**			
Glucuronidase, beta	hGUSB	CTCATTTGGAATTTTGCCGATT	CCGAGTGAAGATCCCCTTTTTA
