# Supplementary material for: Hepatic Wnt1 Inducible Signaling Pathway Protein 1 (WISP-1/CCN4) Associates with Markers of Liver Fibrosis in Severe Obesity
Source: Cells. 2021 Apr 29;10(5):1048. doi: 10.3390/cells10051048 (PMC8146455; doi:10.3390/cells10051048)
Supplement: Supplementary file 1 [file cells-10-01048-s001.zip › Supplement/Table_S2.pdf]

Supplementary Table 2: Correlation analysis of hepatic fibrosis marker and ECM turnover enzymes.

Marker		COL3A1 mRNA	COL6A1 mRNA	αSMA mRNA	MMP9 mRNA	TGFB1 mRNA	TIMP1 mRNA	αSMA staining,%	Sirius red staining,%	Trichrome staining, %
**COL1A1 mRNA**	r	**0.819****[**]**	**0.667**[**]	**0.619**[**]	**0.546**[**]	**0.610**[**]	**0.751**[**]	**0.426**[*]	0.027	0.288
	p	**$5.8x10^{-9}$**	**$2.3x10^{-5}$**	**$1.2x10^{-4}$**	**0.001**	**$1.7x10^{-4}$**	**$4.7x10^{-7}$**	**0.013**	0.883	0.105
**COL3A1 mRNA**	r		**0.729**[**]	**0.484**[**]	**0.619**[**]	**0.672**[**]	**0.638**[**]	0.277	-0.085	0.070
	p		**$1.6x10^{-6}$**	**0.004**	**$1.2x10^{-4}$**	**$1.9x10^{-5}$**	**$6.5x10^{-5}$**	0.119	0.639	0.701
**COL6A1 mRNA**	r			**0.525**[**]	**0.439**[*]	**0.864**[**]	**0.607**[**]	0.285	-0.063	0.026
	p			**0.002**	**0.011**	**$9.8x10^{-11}$**	**$1.8x10^{-4}$**	0.108	0.728	0.886
**αSMA mRNA**	r				**0.369**[*]	**0.573**[**]	**0.626**[**]	**0.420**[*]	0.009	0.079
	p				**0.035**	**$4.9x10^{-4}$**	**$9.8x10^{-5}$**	**0.015**	0.960	0.662
**MMP9 mRNA**	r					**0.524**[**]	**0.484**[**]	0.157	-0.054	0.331
	p					**0.002**	**0.004**	0.384	0.766	0.060
**TGFB1 mRNA**	r						**0.620**[**]	0.255	-0.177	-0.099
	p						**$1.2x10^{-4}$**	0.152	0.323	0.584
**TIMP1 mRNA**	r							**0.433**[*]	0.026	0.148
	p							**0.012**	0.887	0.412
**αSMA staining,%**	r								0.018	0.021
	p								0.917	0.903
**Sirius red staining,%**	r									**0.702**[**]
	p									**$2.6x10^{-6}$**

The data are presented as Pearson's r- and p-values Variables with a skewed distribution were log-transformed prior to the analysis. Histological staining was quantified as a percentage of the whole area using an automated histological image analysis.
